# *Treponema pallidum* (syphilis) antigen TpF1 induces angiogenesis through the activation of the IL-8 pathway

**DOI:** 10.1038/srep18785

**Published:** 2016-01-05

**Authors:** Tommaso Pozzobon, Nicola Facchinello, Fleur Bossi, Nagaja Capitani, Marisa Benagiano, Giulietta Di Benedetto, Cristina Zennaro, Nicole West, Gaia Codolo, Marialina Bernardini, Cosima Tatiana Baldari, Mario Milco D’Elios, Luca Pellegrini, Francesco Argenton, Marina de Bernard

**Affiliations:** 1 Department of Biology, University of Padua, Padua, Italy; 2 Department of Medicine, Surgery and Health Sciences, University of Trieste, Trieste, Italy; 3 Department of Life Sciences, University of Siena, Siena, Italy; 4 Department of Experimental and Clinical Medicine, University of Florence, Florence, Italy; 5 Institute of Neuroscience, Italian National Research Council (CNR), Padua, Italy; 6 Venetian Institute of Molecular Medicine, Padua, Italy; 7 Department of Biology and Biotechnology, Sapienza University of Rome, Rome, Italy; 8 Department of Molecular Biology, Medical Biochemistry and Pathology, Université Laval, Quebec, Canada

## Abstract

Over 10 million people every year become infected by *Treponema pallidum* and develop syphilis, a disease with broad symptomatology that, due to the difficulty to eradicate the pathogen from the highly vascularized secondary sites of infection, is still treated with injections of penicillin. Unlike most other bacterial pathogens, *T. pallidum* infection produces indeed a strong angiogenic response whose mechanism of activation, however, remains unknown. Here, we report that one of the major antigen of *T. pallidum*, the TpF1 protein, has growth factor-like activity on primary cultures of human endothelial cells and activates specific T cells able to promote tissue factor production. The growth factor-like activity is mediated by the secretion of IL-8 but not of VEGF, two known angiogenic factors. The pathogen’s factor signals IL-8 secretion through the activation of the CREB/NF-κB signalling pathway. These findings are recapitulated in an animal model, zebrafish, where we observed that TpF1 injection stimulates angiogenesis and IL-8, but not VEGF, secretion. This study suggests that the angiogenic response observed during secondary syphilis is triggered by TpF1 and that pharmacological therapies directed to inhibit IL-8 response in patients should be explored to treat this disease.

Syphilis is a sexually transmitted disease that is caused by the spirochetal bacterium *Treponema pallidum*. It affects over 10 million people every year and in the past 15 years its spread has increased in North America and in Central and Eastern Europe^1^.

Syphilis is a multistage disease; after the first stage in which a red papule appears and ulcerates the site of inoculation, the bacterium penetrates through the genital mucosa and enters the lymphatic and blood stream, to disseminate into other organs and cause a plethora of clinical manifestations typically defined as secondary syphilis; however, the most common manifestation of secondary syphilis is a disseminated muco-cutaneous rash, characterized by vascular inflammation and increased angiogenesis^2^,^3^. It is widely assumed that angiogenesis could have a crucial role in syphilis pathogenesis for two main reasons. First, the bacterium has limited metabolic capabilities^4^; this implies that the pathogen requires support from the host to derive essential nutrients; second, the microorganism could take advantage of the vascular leakage to gain access to the bloodstream and spread to other parts of the patient’s body.

Compared to normal skin, highly vascularized cutaneous lesions from patients with diagnosed secondary syphilis show that the level of expression of the vascular endothelial growth factor (VEGF) is only minimally affected^5^. This suggests that VEGF may not be the major factor involved in the *T. pallidum*-induced angiogenesis. This observation is not surprising: although the VEGF family has long been considered crucial in the regulation of angiogenesis, other factors have been identified as pro-angiogenic, including chemokines belonging to the CXC family such as CXCL2 and IL-8/CXCL8^6^. These chemokines could be produced either by endothelial cells or by resident and infiltrating inflammatory cells, such as macrophages that accumulate in the muco-cutaneous lesions of secondary syphilis^7^. To date, however, the factor that stimulates the proliferation of endothelial cells and the formation of new blood vessels remains unknown. Thus, the search for the pathogenic factor that triggers angiogenesis in syphilis patients remains an outstanding goal in translational medicine due to its potential as a therapeutic target.

Infiltration of inflammatory cells in the wall of the vessels around the lesions is a histological change that also characterizes tertiary syphilis; typically, the lesions at this stage can degenerate to extensively damage the arterial wall, or to cause atheromatosis. Indeed, relative common causes of death in patients with inadequately treated tertiary syphilis are aneurysm and thrombi that grow on the atherosclerotic plaques^8^. Again, the pathogenic factor that triggers this response is not known.

The fact that syphilis is a chronic infection probably reflects the ability of the bacterium to elicit a T regulatory response, which could be associated with fading of the host effector immune response against the pathogen^9^. We have recently demonstrated that TpF1, a major antigen of *T. pallidum*, plays a pivotal role in driving this suppressive immune response by modulating the release of specific cytokines by monocytes^10^. TpF1 is a protein that shares homology with another immunomodulant antigen, the neutrophil activating protein (HP-NAP), produced by the bacterium *Helicobacter pylori*. Both these proteins belong to the DNA-binding proteins from starved cells (Dps)-like family, a group of bacterial proteins with a dodecameric structure^11^. Similar to HP-NAP, TpF1 interacts with neutrophils and monocytes to modulate their activity^10^,^12^.

In this study, we used *in vitro*, cell based and zebrafish genetics approaches to interrogate the role of TpF1 in syphilis. Our data show that TpF1 stimulates proliferation and migration of endothelial cells and that this effect depends on IL-8 secretion. In addition, we mapped the pathway that activates IL-8 secretion and proved the angiogenic activity of TpF1 in an animal model system, zebrafish. We also report that TpF1 elicits a specific T cell response in syphilis patients: such a response exerts a helper function for endothelial cells that acquire a pro-inflammatory profile and release tissue factor (TF), the molecule that triggers the coagulation cascade.

## Results

### Recombinant TpF1 has growth factor-like activity

**Figure 1:**
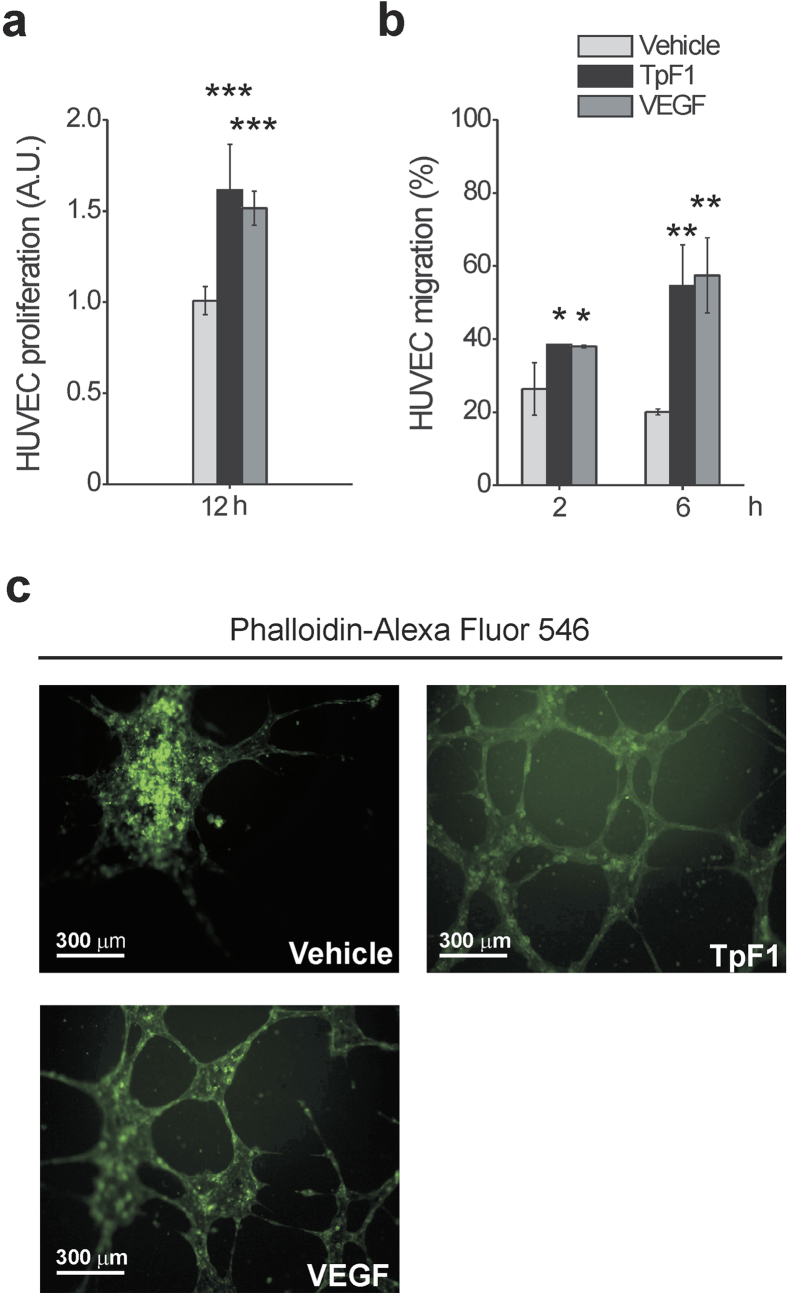
TpF1 induces proliferation and migration of endothelial cells and their organization in microcapillary-like structures. (**a**) HUVECs were exposed to TpF1, VEGF (positive control) or vehicle (saline). After 12 h, cells were counted and normalized to the number of plated cells set as 1 arbitrary unit (A.U.). The graph shows cell proliferation under the different conditions. Data are represented as the mean ± S.D. of three independent experiments. (**b**) Fast DiI-labelled HUVECs were seeded on the upper chambers of 24-transwell plates. TpF1, VEGF, or vehicle, were added to the lower chambers. Migrated cells were quantified after 2 and 6 h by a microplate reader and expressed as % of seeded cells. Data are presented as mean ± S.D. of three independent experiments. (**c**) HUVECs were seeded on Matrigel-coated coverslips and exposed to TpF1, VEGF or vehicle, for 12 h. Cells were stained with Phalloidin-Alexa Fluor 546 and analyzed by confocal microscopy at 200 × magnification. Scale bar = 300 μm.

We started to investigate the growth factor-like activity of TpF1, a major antigen expressed by *T. pallidum*^10^, by comparing the proliferative rate of human umbilical vein endothelial cells (HUVECs) exposed to recombinant TpF1 or VEGF (free of Gram-positive and Gram-negative bacterial contaminants; Suppl. Fig. S1). Data showed that TpF1 stimulates cell proliferation at a rate that is comparable to that of VEGF (Fig. 1a). We then moved to compare the effect on cell migration by using a Transwell model system. Again, results showed a pro-migratory effect of TpF1 similar to that of VEGF (Fig. 1b). We next evaluated the ability of TpF1 to induce the tubular organization that is typically observed upon VEGF stimulation and that is widely accepted to reflect the acquisition of a vessel-like phenotype^13^. Unlike control cells, in this assay, both VEGF and TpF1 stimulate the cells to elongate and to develop spatially organized connections (Fig. 1c). We conclude that local secretion of bacterial TpF1 *in vivo* might have VEGF growth factor-like activity in endothelial cells.

### TpF1 activity is mediated by IL-8

**Figure 2:**
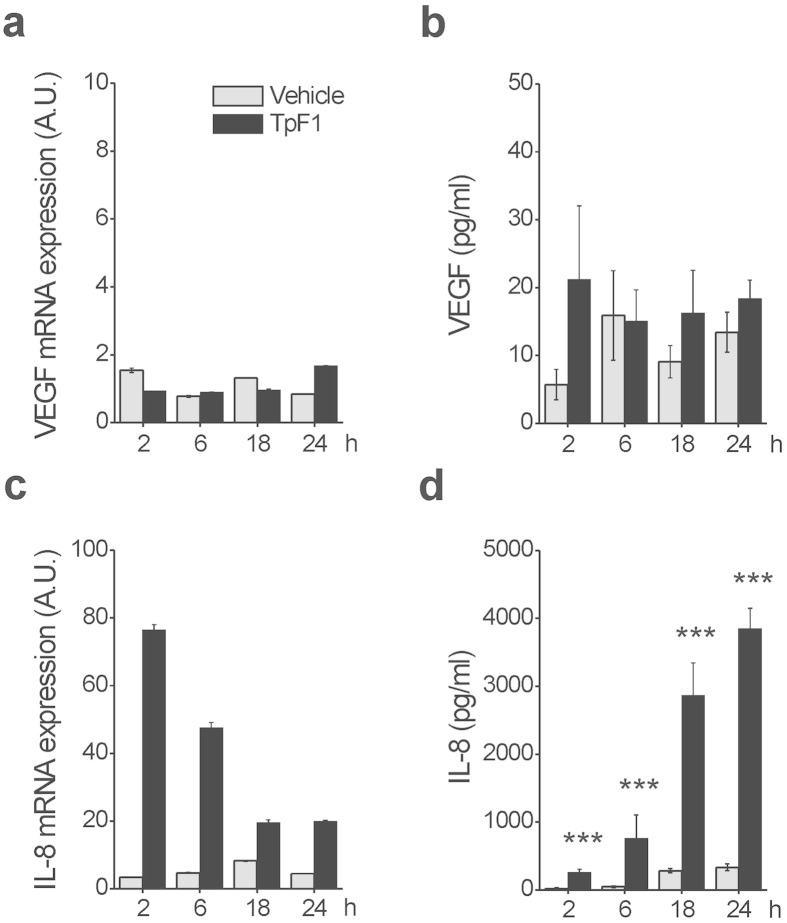
TpF1 stimulates IL-8 expression in endothelial cells. HUVECs were exposed to TpF1 or vehicle (saline) for 2, 6, 18, 24 h and the expression of VEGF (**a**) or IL-8 (**c**) was evaluated by RT-PCR. Data were normalized to an endogenous reference gene (ribosomal subunit 18S). Values at T_0_ cells were taken as reference and set as 1 A.U. and the expression levels for treated cells were relative to the expression of T_0_ cells. Culture supernatants from HUVECs, harvested for quantification of mRNA, were collected and the VEGF (**b**) and IL-8 (**d**) protein content was quantified by ELISA. Data are expressed as mean ± S.D. of four independent experiments. Significance was determined by Student’s *t*-test for data of TpF1 treated cells versus vehicle-exposed cells. ***p < 0.001.

One possible mechanism that could explain the VEGF-like activity of TpF1 is that the bacterial factor could induce VEGF expression *per se* which, in turn, would activate endothelial cell proliferation, migration, and angiogenesis-like differentiation (Fig. 1). To test this possibility we analyzed by RT-PCR and ELISA assay the expression of VEGF in HUVECs stimulated with recombinant TpF1. Data showed that VEGF transcript and protein level do not change upon TpF1 stimulation (Fig. 2a,b), thereby indicating that TpF1 does not activate the VEGF pathway. Therefore, we next tested the mRNA and protein expression level of IL-8. Data showed a rapid and large increase in IL-8 mRNA expression and protein secretion (Fig. 2c,d), suggesting that this chemokine acts as the effector molecule of the putative angiogenic function of TpF1.

**Figure 3:**
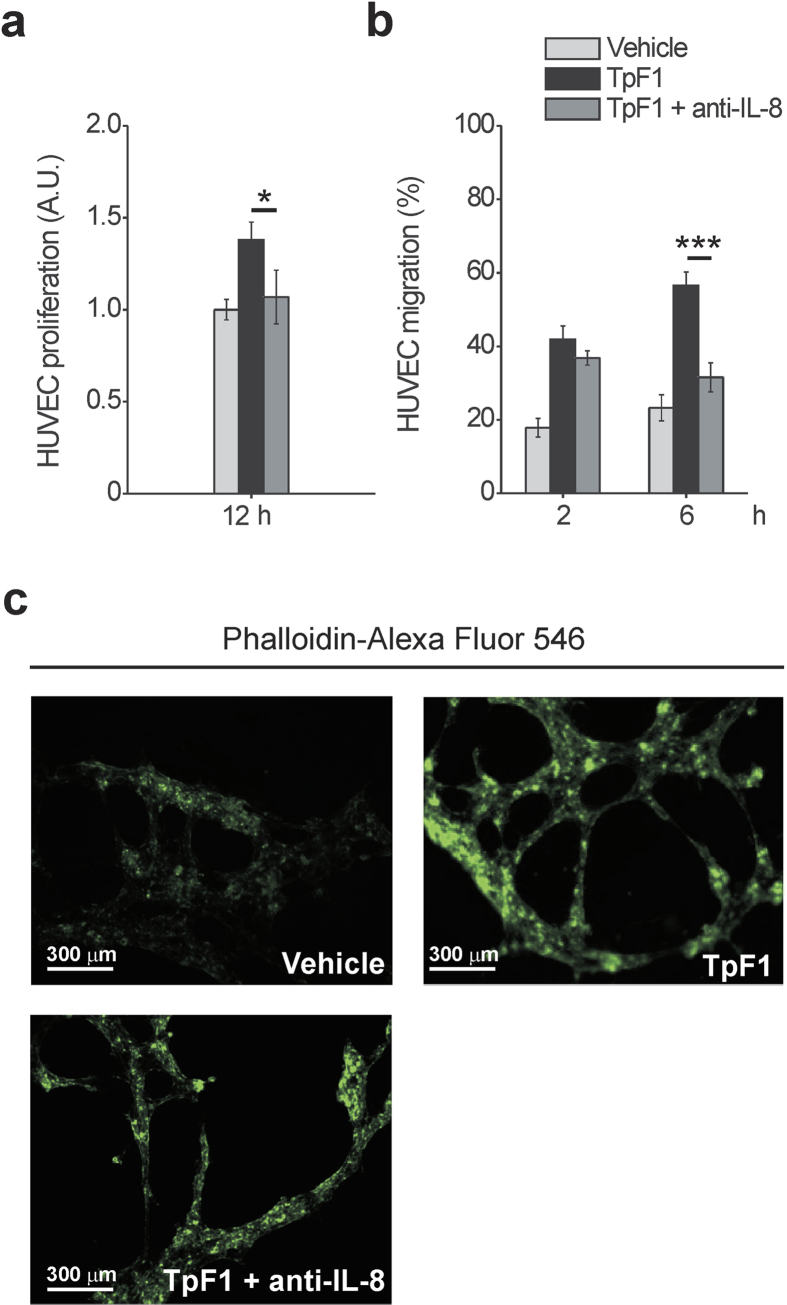
IL-8 is required for the growth factor-like activity of TpF1. (**a**) HUVECs were exposed to TpF1, as in Fig. 1a, in the presence or absence of 1 μg/ml anti-IL-8 blocking antibody. After 12 h cells were counted and normalized to the number of plated cells set as 1 A.U. The graph shows cell proliferation under the different conditions. Data are represented as the mean ± S.D. of three independent experiments. (**b**) HUVECs, grown to confluence in 6-well plates, were exposed to TpF1 for 24 h. Supernatants were collected and incubated or not with anti-IL-8 blocking antibody for 45 min, before transferring to the lower chamber of 24 Transwell plates with Fast DiI-labelled HUVECs seeded on the upper chamber. Migrated cells were quantified after 2 and 6 h. Data are represented as the mean ± S.D. of three independent experiments. (**c**) HUVECs seeded on Matrigel-coated coverslips were exposed to TpF1 for 12 h, in the presence or absence of the IL-8 blocking antibody. Cells were stained with Phalloidin-Alexa Fluor 546 and analyzed by confocal microscopy at 200 × magnification. Scale bar = 300 μm.

To investigate the role of IL-8 in TpF1-induced growth factor-like activity we blocked IL-8 signalling by adding a neutralizing antibody to the media of HUVECs receiving recombinant TpF1. As shown in Fig. 3a, the IL-8-blocking antibody abrogated cell proliferation. Similarly, TpF1-induced cell migration and tubular organization were also abolished by the neutralizing IL-8 antibody (Fig. 3b,c), thereby supporting a model where the cytokine activates a signalling pathway that, ultimately, leads to the vascularization of the tissue infected by the pathogen. Notably, the IL-8-dependent growth factor-like activity of TpF1 was confirmed in dermal microvascular endothelial cells (Suppl. Fig. S2).

### TpF1 signals through the activation of the CREB/NF-κB pathway

**Figure 4:**
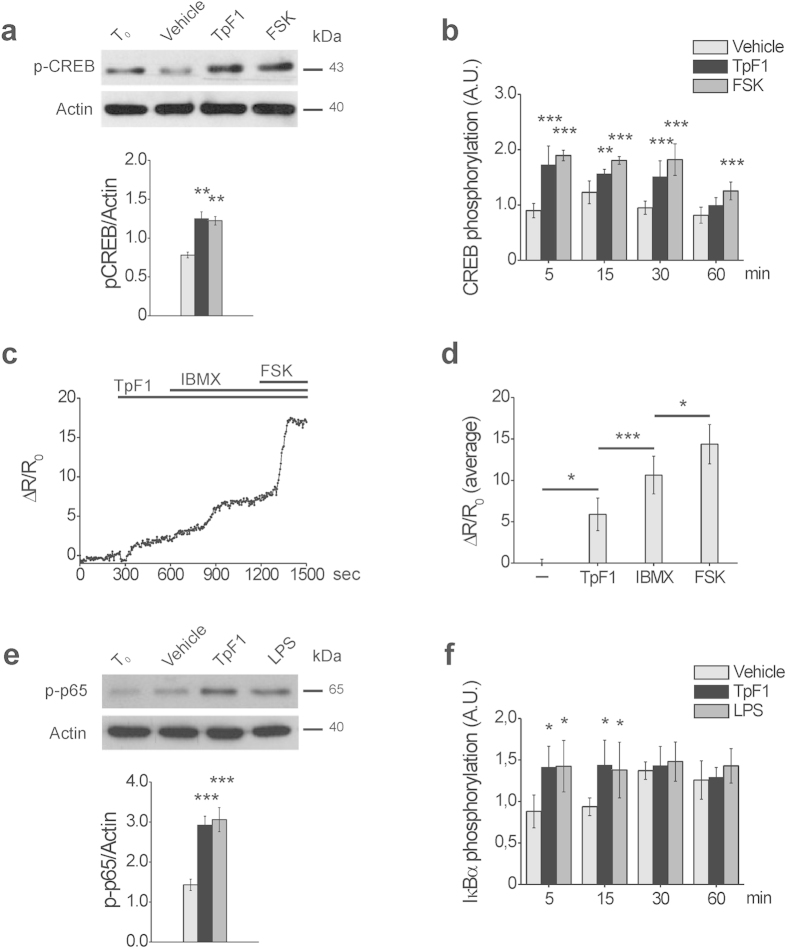
TpF1 activates CREB and NF-κB in endothelial cells. (**a**) HUVECs were exposed to TpF1, forskolin or vehicle (saline). After 30 min, cells were processed for western blot and phospho-CREB and actin were revealed with specific antibodies. The graph shows the ratio between phospho-CREB and actin (n = 4). **p < 0.01. (**b**) HUVECs were exposed to TpF1, forskolin or vehicle. At the indicated time points, the phosphorylation of CREB was evaluated by ELISA assay. For each sample, data were normalized to the total CREB protein. Normalized fluorescence value of cells at time 0 (T_0_) was taken as reference and set as 1 A.U. and CREB phosphorylation of treated cells was expressed as fold change of T_0_ cells. Data are expressed as mean ± S.D. of three independent experiments. Significance was determined by Student’s *t*-test for data of agonist-treated cells versus vehicle-exposed cells. **p < 0.01; ***p < 0.001. (**c**) Representative kinetics of cAMP changes recorded in a Epac1-expressing HUVEC, upon addition of 20 μg/ml TpF1, followed by 100 μM IBMX and by 25 μg/ml forskolin. cAMP variations are presented as ΔR/R_0_. The average ΔR/R_0_ increases (mean ± S.E.M., n = 9) are presented in (**d)**. *p < 0.05; ***p < 0.001. (**e**) HUVECs were exposed to TpF1, LPS or vehicle. After 30 min, cells were processed for western blot. Phospho-p65 and actin were revealed with specific antibodies. The graph shows the ratio between phospho-p65 and actin (n = 4). ***p < 0.001. (**f**) HUVECs were exposed to TpF1, LPS or vehicle. At the indicated time points the phosphorylation of IkB-α was evaluated by ELISA. For each sample, data were normalized to the GAPDH protein. Normalized fluorescence value of cells at time 0 (T_0_) was taken as reference and set as 1 A.U. and IkB-α phosphorylation of treated cells was expressed as fold change of T_0_ cells. Data are expressed as mean ± S.D. of three independent experiments. Significance was determined by Student’s *t*-test for data of agonist-treated cells versus vehicle-exposed cells. *p < 0.05.

It has been shown that the expression of IL-8 involves the activation of CREB and NF-κB^14^. We reasoned, therefore, that the growth factor-like activity of TpF1, which ultimately should result in *de novo* vascularization, might be mediated by the activation of these two transcription factors. To address this possibility, first we monitored CREB activation by looking at the phosphorylation state of its Ser133 residue^15^. More specifically, we compared phospho-CREB levels in HUVECs stimulated with recombinant TpF1 and, as positive control, with forskolin, a known specific activator of CREB^16^,^17^. Data indicates that TpF1 stimulates CREB phosphorylation with a similar kinetic of that induced by forskolin (Fig. 4a,b). Since CREB activation depends on a raise in intracellular cAMP level^16^, we validated this observation measuring by FRET analysis changes in cAMP. As expected, TpF1 induced a raise in the level of the second messenger (Fig. 4c). Noteworthy, using well-established pharmacological tools in cAMP analysis, we estimated this raise to account for the 30% of the maximal potential cAMP increase possible for HUVECs (Fig. 4d); this value is in line with that reported for other CREB activators^18^,^19^.

Lastly, we investigated the activation of NF-κB in TpF1-stimulated HUVECs by monitoring the phosphorylation state of p65 as well as that of IκBα^20^. We found that the response of the cells to TpF1 overlapped that of cells stimulated with LPS (Fig. 4e,f), a known activator of NF-κB^21^. Altogether, these data indicate that TpF1 triggers IL-8 via the activation of the CREB/NF-κB pathway.

### CREB/NF-κB activation mediates TpF1-induced IL-8 secretion

**Figure 5:**
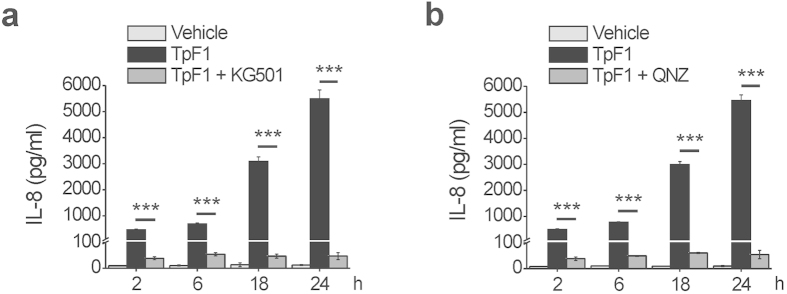
CREB and NF-κB are essential for the production of IL-8 by endothelial cells exposed to TpF1. HUVECs were pre-incubated, or not, for 30 min with the CREB inhibitor KG-501 (**a**) or the NF-κB inhibitor QNZ (**b**), before the exposure to TpF1 or vehicle. At the indicated time points, culture supernatants were collected and evaluated for their IL-8 content by ELISA. Data are expressed as mean ± S.D. of three independent experiments. ***p < 0.001.

Next, we functionally linked CREB/NF-κB activation to TpF1-induced IL-8 secretion. 2-naphthol-AS-E-phosphate (KG-501) is a small molecule that binds to the transcription co-activator CREB-binding protein (CBP) and blocks its interaction with the active form of CREB, phospho-CREB^22^. N4-[2-(4-phenoxyphenyl)ethy-4,6-quinazolinediamine (QNZ) is an antagonist of the NF-κB activation pathway, acting by inhibiting store-operated calcium (Ca^2+^) entry^23^. When added to HUVECs prior to TpF1 stimulation either inhibitor effectively blocked IL-8 secretion (Fig. 5a,b), thereby supporting the notion that TpF1 is a specific activator of this chemokine.

### TpF1 has angiogenic activity in zebrafish

Collectively, our data suggest that TpF1 is the angiogenic factor of *T. pallidum* that, through the stimulation of IL-8 during secondary syphilis, causes vascularization of the infected tissues. However, this possibility cannot be tested directly for three reasons. First, *T. pallidum* is not a genetically amendable microorganism^3^. Second, humans are the pathogen’s only natural host; the experimental transmission of syphilis to a number of animals, including primates, has been attempted, but only subcutaneous injection of the pathogen in rabbits has proved to be somewhat successful^8^. Third, rabbits infected with *T. pallidum* develop primary lesions that are essentially identical to those seen in humans, but not the secondary vascularised lesions^8^. Thus, considering these limitations, we moved to address TpF1 angiogenic activity *in vivo* taking advantage of an animal model that has been widely used to study mammalian angiogenesis: zebrafish. In zebrafish, human VEGF drives blood vessel formation^24^, therefore this animal is a useful model to interrogate the angiogenic activity of *T. pallidum* TpF1.

**Figure 6:**
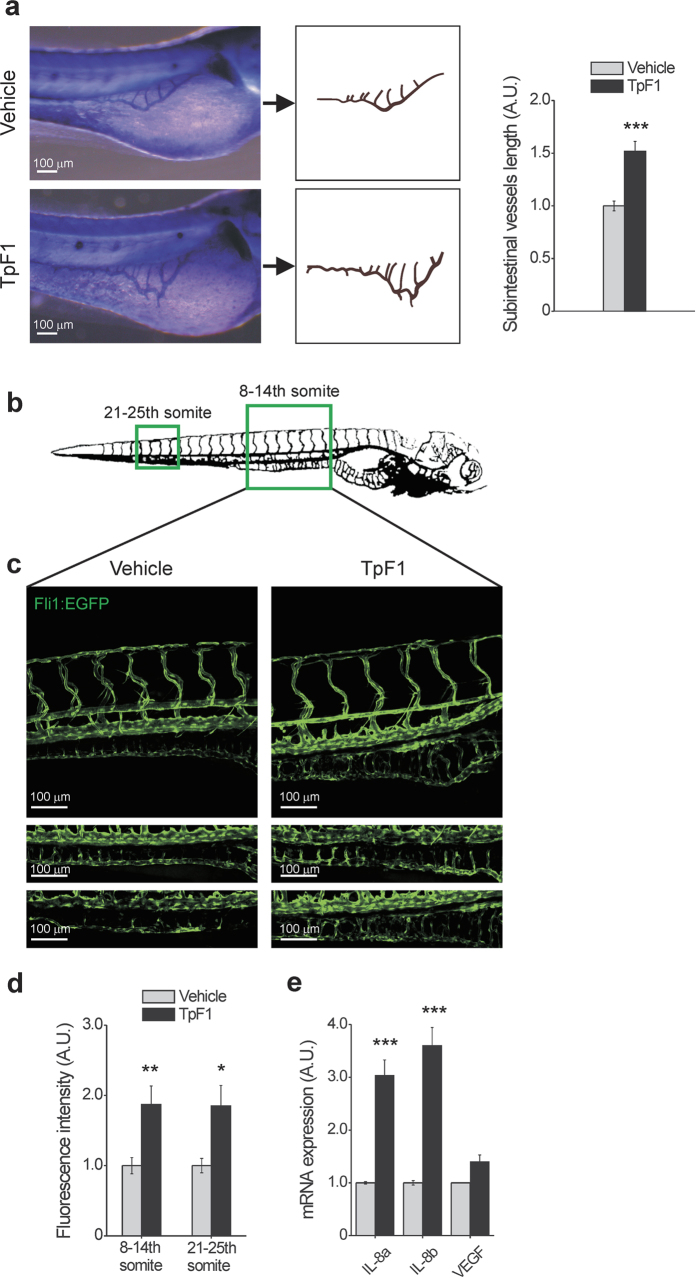
TpF1 has angiogenic activity in zebrafish. (**a**) Comparison of the blood vessel patterning, at the sub-intestinal area (SIVs), between vehicle- and TpF1-treated larvae. Animals were injected in the yolk 1 dpf and the alkaline phosphatase-based assay was performed at 3 dpf. Scale bar = 100 μm. Graph represents the fold increase in SIV length referring to the value of vehicle-treated animals, set as 1. Data are expressed as mean ± S.E.M. (n = 20 for each treatment). Significance was determined by Student’s *t*-test for data of TpF1-treated fish versus vehicle-exposed fish. ***p < 0.001. (**b**) Illustration of the vascular system of a 4 dpf larva. Regions for image acquisition are indicated by boxes. (**c**) Embryos at 2 dpf were injected in the yolk or in the caudal vein plexus with TpF1 or vehicle. Representative images were taken at 4 dpf by confocal microscopy at 20 × magnification. Scale bar = 100 μm. (**d**) Integrated density of fluorescence in the green channel was determined in the segment between 8 and 14th and between 21st and 25th somite. Values of vehicle-treated animals were set as 1 A.U. and used as reference for TpF1-treated animals (mean ± S.E.M., n = 6). Significance was determined by Student’s *t*-test for data of TpF1-treated fish versus vehicle-exposed fish. *p < 0.05; **p < 0.01. (**e**) Embryos were injected in the yolk with TpF1 or vehicle at 2 dpf. RNA was extracted at 4 dpf and the expression of VEGF, IL-8a and IL-8b was evaluated by RT-PCR. For each sample, data were normalized to the endogenous reference gene β-actin. Vehicle-treated fish were taken as reference and set as 1 A.U. Expression levels of TpF1-exposed fish were expressed as fold change relative to the expression level of vehicle-treated fish. Data represent mean ± S.E.M. (n = 30 larvae for each treatment). Significance was determined by Student’s *t*-test for data of TpF1-treated fish versus vehicle-exposed fish. ***p < 0.001.

As an initial assay, we injected recombinant TpF1 in the yolk of zebrafish larvae (1 day post fertilization; n = 20) and, two days later, measured the length of their sub-intestinal veins^24^. Data showed a nearly 50% increase of the overall vascularization of the abdomen (Fig. 6a). Next, we injected TpF1 in either the yolk sac or in the caudal plexus of larvae of transgenic zebrafish that express GFP in the endothelial cells of blood vessels (2 days post fertilization; n = 6)^25^, and measured the impact of TpF1 on the intestinal vascularization of the 8–14th and 21–25th somites of the fish (boxed areas in Fig. 6b). As expected, we found that TpF1 increased the overall level of GFP expression as well as the number of cells expressing GFP (Fig. 6c,d), which, together, indicate the formation of new blood vessels. In these animals, we also observed an increased expression of IL-8, but not of VEGF (Fig. 6e), thereby confirming the role of this cytokine in mediating the angiogenic activity of TpF1.

### Syphilis patients have TpF1-specific T cells that trigger the secretion of IL-8, CCL-20 and tissue factor

The evidence that syphilis patients have antibodies against TpF1, prompted us to search for T cells that are specific for this factor and to evaluate its contribution in the aetiology of the disease.

We found that patients affected by tertiary syphilis possess TpF1-specific T cells and that these cells, once activated by TpF1, but not by the control antigen (tetanus toxoid, data not shown), stimulate HUVECs to secrete IL-8 and CCL-20 (Table 1). These chemokines promote vascular inflammation and play a pathogenic role in the development of atherosclerotic lesions^26^,^27^, suggesting a role of TpF1 in the development of these late symptoms of the disease.

Similar data were obtained with monocytes, cells that infiltrate the endothelia in the syphilis lesions and are recruited during the formation of the atherosclerotic plaques (Table 2). No production of other angiogenic factors, such as CCL-1, CCL-13, angiopoietin-1, angiopoietin-2 was found by both HUVECs and monocytes, after stimulation by freshly TpF1-activated T cells.

To address weather TpF1-specific T cells might also contribute to the formation of trombi on the plaque, we tested weather TpF1-specific T cells could trigger the release of tissue factor (TF) in HUVECs and monocytes. Data show that TpF1-specific T cells following stimulation with TpF1, but not with tetanus toxoid (data not shown), lead to the secretion of TF in both types of primary cells (Tables 1 and 2).

Taken together, these findings indicate that TpF1 activates T lymphocytes in syphilis patients; these cells stimulate the pro-inflammatory and pro-coagulant function of endothelial cells, thereby supporting the possibility that they might contribute to the pathogenesis of the vascular inflammatory lesions of the late stage of the disease.

## Discussion

Angiogenesis is associated with physiological events, such as pregnancy, organ formation, and wound repair, and constitutes a major pathological determinant in the growth of solid tumours. Bacterial infections can also induce angiogenesis but, in humans, only few bacteria stimulate angiogenesis; it is not surprising, therefore, that vascularised lesions are considered a hallmark diagnostic symptom of the microorganisms responsible for the underlying diseases. Among them is *Bartonella bacilliformis*, a proteobacterium responsible for verruga peruana (Carrion’s disease). Its infection causes tumour-like angiomatous lesions on the patients’ skin through the expression of two angiogenic factors, angiopoietin-2 and VEGF^28^. *T. pallidum* is another bacterium that produces similar symptoms. In this case, however, the lesions appear to be caused by the expression of a chemokine, IL-8, indicating that different bacteria trigger vascularization through the activation of distinct angiogenic pathways in the host.

The involvement of IL-8 in bacteria-associated angiogenesis has also been reported for *Bartonella henselae*. However, here the type of vascularization produced by this chemokine is very different from that observed in patients infected by *T. pallidum*. Indeed, whereas *B. henselae* causes a type of microvascularization of the skin that is reminiscent of that observed in a neoplasia^29^, *T. pallidum* leads to the formation of blood vessels that form a more complex and diverse type of vascular network. This suggests that the type of vascularization triggered by either *B. henselae* or *T. pallidum* depends on the pathway downstream of IL-8, rather than on the activation of IL-8 *per se*.

Vascular inflammation characterizes the lesions of secondary and tertiary syphilis and contributes to the degenerative symptoms that accompany the late stages of the disease. T cells cooperate to the maintenance of this process by homing at the sites of inflammation and influence the physiology of resident and recruited cells, such as the endothelial cells and monocytes that we used in this study^30^,^31^. We prove that syphilis patients have TpF1-specific T cells, and that they can activate the secretion of those factors that are known to sustain the inflammatory response (Tables 1 and 2). These findings support a model where TpF1-specific T cells home to the sites of *T. pallidum* infection, to activate a response that, through the expression of IL-8, CCL-20 and TF, contributes to the exacerbation of the syphilis patients symptoms.

Collectively, our results identify TpF1 as a *T. pallidum* factor relevant for all symptoms of the secondary and tertiary stage of syphilis, namely vascular inflammation, angiogenesis and cardiovascular complications. Our findings also suggest that therapeutic strategies aimed at inhibiting IL-8 activity could be employed against it. A fully human monoclonal antibody against IL-8 has been developed and is currently under investigation for the treatment of chronic inflammatory diseases^32^. Its use could also be considered as a therapeutic approach for syphilis.

## Material and Methods

### Reagents

HEPES, human endothelial serum-free medium (HE-SFM), Fast DiI, Phalloidin-Alexa Fluor 546, Trizol Reagent, Superscript II, MMLV reverse transcriptase and protease inhibitor cocktail were from Life Technologies (Carlsbad, CA, USA); Medium 199 (M199), foetal bovine serum (FBS), heparin, forskolin, LPS, Tricaine, phytohemoagglutinin (PHA) and 1-phenyl-2-thiourea were from Sigma-Aldrich (Saint. Louis, MO, USA); L-glutamine, trypsin-EDTA, penicillin-streptomycin, were from Euroclone (Siziano, IT). Collagenase was from Worthington Biochemical Corporation (Lakewood, NJ, USA). Fibronectin was from Roche (Basilea, Switzerland). Matrigel was from Becton, Dickinson & Company (Frankin Lakes, NJ, USA). Goat anti-human IL-8 blocking antibody was from Abcam (Cambridge, UK); human recombinant VEGF-A was from Immunological Sciences (Rome, IT). Monoclonal antibody anti anti-actin (clone C4), KG-501 and QNZ were from Millipore (Billerica, MA, USA); rabbit polyclonal antibodies anti-phospho-CREB (ser133) and anti-phospho-p65 were from Cell Signalling Technology (Danvers, MA, USA).

### Expression and purification of recombinant TpF1

TpF1 was cloned, expressed, and purified from *Bacillus subtilis* to avoid contamination with LPS, as described previously^10^. Briefly, TpF1 expressing bacterial cells were lysed and the protein purified by FPLC on anion-exchange Mono Q columns. Subsequently, the recombinant protein was purified by gel-filtration chromatography on Superdex 200 HR 10/30 columns, and concentrated by Vivaspin ultrafiltration. The purity of TpF1 was 98% as determined by Coomassie gel.

### Testing the absence of Gram-negative and Gram-positive contaminants from recombinant TpF1 preparations

Stably transfected HEK 293 hTLR4/MD2/CD14 cells (InvivoGen, San Diego, CA, USA) were seeded into 96-well plates at the density of 3 × 10^5^ cells/ml. Cells were transfected with Firefly luciferase reporter constructs, pGL3.ELAM.tk, and Renilla luciferase reporter plasmid, pRLTK as published^33^. HEK.293 hTLR4/MD2/CD14 were exposed to 100 ng/ml E. coli LPS (LPS-EB ultrapure, InvivoGen) or to 100 ng/ml TpF1. Cells were stimulated for 4 h and 18 h. Similarly, stably transfected HEK293 hTLR2 cells (InvivoGen) were exposed to 100 ng/ml TpF1 or to 1 μg/ml Pam3CSK4 (InvivoGen). Stimulation was carried out for 6 h and 18 h. NF-κB-dependent luciferase activity was measured at 4 h (for hTLR4/MD2/CD14) and 6 h (for hTLR2) using the Dual-Luciferase Reporter Assay System (Promega, Fitchburg, WV, USA), as reported^33^. IL-8 release was quantified at 18 h of stimulation by ELISA. Absence o bacterial contaminants in recombinant VEGF was tested by the manufacturer (Immunological Sciences).

### Human Umbilical Vein Endothelial Cell (HUVEC) preparation

Umbilical cords, obtained from full-term healthy pregnant women, were anonymously provided by the Hospital of Padua, Italy. Informed consent was obtained by all subjects. Collagenase treatment was performed to isolate HUVECs from the cords and cells were cultured as previously published^34^. Cells were used at passage 2–5.

### Proliferation assay

HUVECs or human dermal microvascular endothelial cells (HMVEC-D, Lonza, Basel, Switzerland) were seeded in 2% gelatin-coated 96-well tissue culture plate at 1 × 10^4^ cells/well and maintained in M199 complete medium for 2 h before the application of stimuli in HE-SFM. Cells were exposed to 20 μg/ml TpF1 or 20 ng/ml VEGF-A or vehicle (saline). For the inhibition experiments, cells were pre-treated with 1 μg/ml anti-IL-8 blocking antibody that was maintained during TpF1 treatment. After 12 h, cells were harvested and counted with a cell counter (Beckman Coulter). The proliferation rate is reported as the fold increase in cell number with respect to the number of plated cells.

### Migration assay

Cell migration was evaluated by Transwell migration assay coating the lower side of the polycarbonate filter (8 μm pores) with fibronectin (5 μg/cm^2^)^13^. Fast DiI-labelled HUVECs or HMVEC-D (2 × 10^5^ cells) were added to the upper compartment in HE-SFM and 20 μg/ml TpF1 or 20 ng/ml VEGF-A, used as positive control, were added to the lower compartment. For the inhibition experiments, supernatant of HUVECs or HMVEC-D, seeded in a 6-well tissue culture plate, grown to confluence and stimulated in HE-SFM with 20 μg/ml TpF1 for 24 h, was pre-incubated with 1 μg/ml anti-IL-8 blocking antibody for 45 min at 37 °C and then added to the lower compartment. Migrated cells were quantified after 2 and 6 h by a microplate reader (Infinite 200, Tecan), comparing to a standard curve and expressed as % of seeded cells.

### *In vitro* formation of microcapillary-like structures

HUVECs or HMVEC-D (5.5 × 10^4^) were seeded on wells coated with Matrigel (12 mg/ml), and incubated for 10 h with 20 μg/ml TpF1, in presence or absence of 1 μg/ml anti-IL-8 blocking antibody. VEGF-A was used as positive control. After fixation with 4% paraformaldehyde and staining with Phalloidin-Alexa Fluor 546, images were obtained using a Leica DM2000 microscope with HC PL Fluotar 20×/0.50 lenses, a Leica DFC 490 digital camera and running the Leica Application Suite software. The number of tubes formed was counted and expressed as number of tubes/cm^2^ 13.

### Isolation of T cells from tertiary syphilis patients and evaluation of their TpF1 specificity

All the experimental protocols were approved by the ethical committee of the Department of Experimental and Clinical Medicine University of Florence, Italy. T cells were obtained from the peripheral blood of 10 patients with tertiary syphilis (5 men, mean age 58; 5 women, mean age 52), after obtaining written informed consent from all patients. The TpF1 specificity of freshly isolated T cells was assessed by measurement of [^3^H]TdR uptake after 60 h of stimulation with 10 μg/ml TpF1 under MHC-restricted conditions^35^. Mitogenic index (ratio of mean cpm of stimulated to unstimulated cultures) >5 was considered as positive. The percentage of TpF1-specific T cells in the peripheral blood of syphilis patients was quite high (0.8% ± 0.15), whereas no TpF1-specific T cell was found in the peripheral blood of healthy subjects (0.002 ± 0.0004).

### RT-PCR analysis on HUVECs

HUVECs were seeded in 2% gelatin-coated 24-well tissue culture plate at 4 × 10^4^ cells/well and maintained in M199 complete medium until they reached 80% confluence. Cells were stimulated with 20 μg/ml TpF1 in HE-SFM, harvested, and total RNA was isolated using RNeasy Kit according to the manufacturer’s instructions (Qiagen). RNA was reverse-transcribed using Superscript II and cDNA was amplified with the following primers: ribosomal subunit 18S, 5′-CGGCTACCACATCCAAGGAA-3′ and 5′-GCTGGAATTAGCGCGGCT-3′; VEGF-A, 5′-GCCTTGCCTTGCTGCTCTA-3′ and 5′-GATGTCCACCAGGGTCTCG-3′; IL-8, 5′-TTGGCAGCCTTCCTGATT-3′ and 5′-AACTTCTCCACAACCCTCTG-3′. After amplification, data analysis was performed using the second derivative algorithm by applying the 2^−ΔΔCT^ method. For each sample, data were normalized to the endogenous reference gene (ribosomal subunit 18S). Cells harvested at time zero were taken as the reference value, set to 1 A.U. (arbitrary unit), and the relative expression levels for treated or untreated cells were calculated and shown.

### ELISA

Culture supernatants of HUVECs, harvested for quantification of mRNAs, were collected and stored at −80 °C for subsequent quantification of cytokine content by ELISA: specific kits for IL-8 and VEGF-A (Raybiotech) were used following the manufacturers’ instructions. When required, cells were pre-incubated for 30 min with 10 μM KG-501 or 100 nM QNZ before the exposure to TpF1.

Freshly isolated T-cells (8 × 10^5^/ml) were co-cultured with HUVECs or autologous monocytes (4 × 10^5^/ml) in the presence of 10 μg/ml TpF1 or 0.5 μg/ml tetanus toxoid (GSK, London, UK). After 24 h, the content of IL-8, CCL-20, TF, CCL-1, CCL-13, angiopoietin-1 and angiopoietin-2 in the culture supernatants was quantified by specific ELISA kits (IL-8: Affymetrix, eBioscience, Santa Clara, CA, USA; CCL-20, CCL-1, angiopoietin-1, angiopoietin-2: R&D Systems, Minneapolis, MN, USA; TF: Sekisui Diagnostics, Pfungstadt, Germany; CCL-13: Thermofisher Scientific, Waltham, MA, USA).

### Evaluation of the phosphorylation state of CREB and NF-κB p65 subunit

HUVECs were seeded in 2% gelatin-coated 6-well tissue culture plate at 3 × 10^5^ cells/well and maintained in M199 complete medium until they reached 80% confluence. Cells were washed with HE-SFM and incubated in the same medium with 20 μg/ml TpF1, 25 μM forskolin, 1 μg/ml LPS or vehicle (saline) for 30 min. Cells were then washed with ice-cold PBS, lysed in 1% Triton X-100, 20 mM Tris-HCl pH 8.0, 150 mM NaCl in the presence of a protease inhibitor cocktail, resolved by SDS-PAGE and transferred to nitrocellulose. Phospho-proteins were revealed by specific polyclonal antibodies. After band densitometry, actin was used to normalize the amount of the phospho-proteins.

### Whole cell ELISA

HUVECs were seeded in 2% gelatin-coated 96-well tissue culture clear-bottom black plate (R&D Systems) at 1 × 10^4^ cells/well and maintained in M199 complete medium until they reached 80% confluence. Cells were exposed for 5, 15, 30 and 60 min to 20 μg/ml TpF1, 1 μg/ml LPS, 25 μM forskolin or vehicle (saline) in HE-SFM. Phosphorylation of CREB on serine 133 and phosphorylation of IkB-α on serine 32 and 36 were evaluated by whole-cell ELISA according to the manufacturer’s instructions (R&D). For each sample, data were normalized to the total CREB protein or to the GAPDH protein in case of IkB-α. The normalized fluorescence value of cells at time 0 was taken as reference and set as 1 A.U.; CREB and IkB-α phosphorylation of treated cells was expressed as fold change with respect to the time 0.

### Fluorescence Resonance Energy Transfer Imaging

HUVECs (5 × 10^5^ cell/well), seeded on 24 mm diameter gelatin-coated coverslips in M199 complete medium 20% FBS, were transfected with 1 μg of mammalian expression plasmid encoding the cAMP biosensor Epac1-camps^36^, using Amaxa Nucleofector Technology according to the manufacturer’s instructions (Basic Nucleofector Kit for Primary Endothelial Cells, Lonza, Basel, Switzerland). FRET imaging experiments were performed 24–48 h after cell transfection. HUVECs were maintained at 37 °C in HEPES-buffered Ringer-modified saline (20 mM Hepes, pH 7.4, 125 mM NaCl, 5 mM KCl, 1 mM Na_3_PO_4_, 1 mM MgSO_4_, 2 mM CaCl_2_, 5.5 mM glucose) and imaged with an inverted microscope (Olympus IX50) equipped with a CellR imaging system and a beam-splitter optical device (Multispec Microimager; Optical Insights). Images were acquired every 5 s with a 63×, 1.4 NA oil-immersion objective (Olympus) using the Cell^R^ software and further processed using ImageJ (http://rsb.info.nih.gov/ij/). FRET changes were measured as changes in the background-subtracted 480/545 nm fluorescence emission intensities upon excitation at 430 nm and expressed as ΔR/R_0_, where R is the ratio at time t and R_0_ is the ratio at time = 0 s; ΔR = R–R_0_.

### Zebrafish injection and imaging

Zebrafish handling and treatments were approved by the Ethical Committee on Animal Experimentation of the University of Padua (CEASA - Project #19/2010) and methods were carried out in accordance with the approved guidelines. Embryos were obtained from natural spawning of wild-type and Tg(Fli1:EGFP)^y1^ transgenic zebrafish (*Danio rerio*)^25^.

For Sub-Intestinal Vein observation (SIV assay), wild-type zebrafish embryos were injected with 200 μg/ml of TpF1 at 1 day post fertilization (dpf) before being fixed in 4% paraformaldehyde (PFA) at 3 dpf and stained for endogenous alkaline phosphatase activity according to the method described in Serbedzija and colleagues^24^. Images were obtained with a digital camera (Leica DFC 295) coupled to a stereomicroscope Leica S8PO (80 × total magnification) using Leica Application Suite Software. The measure of SIV length was determined using Sigma Scan Pro software.

Embryos from Tg(Fli1:EGFP)^y1^ transgenic zebrafish were raised and fish were maintained as described^37^. Embryos were treated with 1-phenyl-2-thiourea to inhibit pigment formation^37^. For TpF1 injection, anesthetized living embryos at 2 dpf were embedded in low-melting agarose and injected under a dissecting stereomicroscope via glass capillaries. Larvae were injected in the yolk or in the caudal vein with 200 μg/ml of TpF1 or vehicle (saline). At 4 dpf larvae were anesthetized using Tricaine and mounted in 0.8% low melting agarose on a glass lid before photographing. Images were obtained using a C2 Nikon confocal microscope with Nikon Fuor 20×/0.50W lenses, running an imaging software NIS Elements 4.0 (Nikon). The integrated density of fluorescence of vessels (integrated sum of fluorescence level for each pixel) was calculated using VOLOCITY 6.0 software (Perkin Elmer) on GFP-positive confocal acquired images.

### RNA Isolation and RT-PCR in zebrafish

A group of 30 larvae were injected at 2 dpf into the yolk extension with 200 μg/ml TpF1 and a second group of 30 larvae were injected with vehicle (saline). At 4 dpf, RNA was extracted from the larvae of each group, using Trizol Reagent, and pooled. One microgram of total RNA was reverse transcribed using MMLV reverse transcriptase and cDNA was amplified with the following primers: *Danio rerio* β-actin, 5′*-*CAGCAAGCAGGAGTACGATGAGT-3′ and 5′-TTGAATCTCATTGCTAGGCCATT-3′; *Danio rerio* IL-8a, 5′-AGCTTGAGAGGTCTGGCTGTAGA-3′ and 5′-CGAGGCGTTGATAAGCTCTCTGCT-3′, *Danio rerio* IL-8b, 5′-TGTTTTCCTGGCATTTCTGACC-3′ and 5′-TTTACAGTGTGGGCT TGGAGGG-3′, *Danio rerio* VEGF-A, 5′-GATGTGATTCCCTTCATGGATGTGT-3′ and 5′-GGATACTCCTGGATG ATGTCTACCA-3′.

### Statistical analysis

Data are reported as the mean ± S.D. or S.E.M., where specifically indicated. Student’s *t* test was used for statistical analysis of the differences between experimental groups. The *p* values ≤ 0.05 were considered significant.

## Additional Information

**How to cite this article**: Pozzobon, T. *et al.* *Treponema pallidum* (syphilis) antigen TpF1 induces angiogenesis through the activation of the IL-8 pathway. *Sci. Rep.* **6**, 18785; doi: 10.1038/srep18785 (2016).

## Supplementary Material

Supplementary Information 1

## Figures and Tables

**Table 1:** TpF1-specific T cells from tertiary syphilis patients stimulate HUVECs to release TF, IL-8, CCL-20.

Syphilis patients	TF pg/ml	IL-8 pg/ml	CCL-20 pg/ml	CCL-1 pg/ml	CCL-13 pg/ml	ANGPT-1 pg/ml	ANGPT-2 pg/ml
1	353 ± 43	142 ± 87	158 ± 112	<8	<8	<8	<8
2	267 ± 21	115 ± 79	89 ± 43	<8	<8	<8	<8
3	186 ± 64	166 ± 91	112 ± 32	<8	<8	<8	<8
4	298 ± 51	122 ± 48	201 ± 24	<8	<8	<8	<8
5	295 ± 43	173 ± 29	184 ± 33	<8	<8	<8	<8
6	338 ± 72	214 ± 47	139 ± 41	<8	<8	<8	<8
7	387 ± 98	278 ± 62	106 ± 37	<8	<8	<8	<8
8	273 ± 60	164 ± 73	139 ± 48	<8	<8	<8	<8
9	382 ± 115	202 ± 76	182 ± 36	<8	<8	<8	<8
10	253 ± 49	177 ± 38	133 ± 25	<8	<8	<8	<8

ANGPT = angiopoietin.

**Table 2:** TpF1-specific T cells from tertiary syphilis patients stimulate monocytes to release TF, IL-8, CCL-20.

Syphilis patients	TF pg/ml	IL-8 pg/ml	CCL-20 pg/ml	CCL-1 pg/ml	CCL-13 pg/ml	ANGPT-1 pg/ml	ANGPT-2 pg/ml
1	368 ± 41	153 ± 71	186 ± 44	<8	<8	<8	<8
2	299 ± 22	134 ± 66	87 ± 56	<8	<8	<8	<8
3	204 ± 64	142 ± 53	126 ± 47	<8	<8	<8	<8
4	311 ± 52	197 ± 43	263 ± 33	<8	<8	<8	<8
5	316 ± 44	182 ± 32	199 ± 41	<8	<8	<8	<8
6	355 ± 75	264 ± 24	154 ± 52	<8	<8	<8	<8
7	392 ± 57	291 ± 73	137 ± 38	<8	<8	<8	<8
8	286 ± 55	177 ± 65	169 ± 55	<8	<8	<8	<8
9	404 ± 67	276 ± 56	189 ± 70	<8	<8	<8	<8
10	278 ± 61	139 ± 49	151 ± 49	<8	<8	<8	<8

ANGPT = angiopoietin.
