# Corrigendum: *Treponema pallidum* (syphilis) antigen TpF1 induces angiogenesis through the activation of the IL-8 pathway

**DOI:** 10.1038/srep46945

**Published:** 2018-02-16

**Authors:** Tommaso Pozzobon, Nicola Facchinello, Fleur Bossi, Nagaja Capitani, Marisa Benagiano, Giulietta Di Benedetto, Cristina Zennaro, Nicole West, Gaia Codolo, Marialina Bernardini, Cosima Tatiana Baldari, Mario Milco D’Elios, Luca Pellegrini, Francesco Argenton, Marina de Bernard

Scientific Reports 6: Article number: 1878510.1038/srep18785; published online: 01 05 2016; updated: 02 16 2018

This Article contains errors in the Materials and Methods section under subheading ‘RNA Isolation and RT-PCR in zebrafish’.

“One microgram of total RNA was reverse transcribed using MMLV reverse transcriptase and cDNA was amplified with the following primers: *Danio rerio* β-actin, 5′-CAGCAAGCAGGAGTACGATGAGT-3′ and 5′-TTGAATCTCATTGCTAGGCCATT-3′; *Danio rerio* IL-8a, 5′-AGCTTGAGAGGTCTGGCTGTAGA-3′ and 5′-CGAGGCGTTGATAAGCTCTCTGCT-3′, *Danio rerio* IL-8b, 5′-TGTTTTCCTGGCATTTCTGACC-3′and 5′-TTTACAGTGTGGGCT TGGAGGG-3′, *Danio rerio* VEGF-A, 5′-GATGTGATTCCCTTCATGGATGTGT-3′ and 5′-GGATACTCCTGGATG ATGTCTACCA-3′.”

should read:

“One microgram of total RNA was reverse transcribed using MMLV reverse transcriptase and cDNA was amplified with the following primers: *Danio rerio* VEGF-A, 5′-GATGTGATTCCCTTCATGGATGTGT-3′ and 5′-GGATACTCCTGGATGATGTCTACCA-3′; *Danio rerio* β-actin, 5′-CAGCAAGCAGGAGTACGATGAGT-3′ and 5′-TTGAATCTCATTGCTAGGCCATT-3′; for the IL-8 gene, amplification of two different fragments was performed with two couples of primers, hereafter be referred to as *a* and *b*: *Danio rerio* IL-8*a*, 5′-AGCTTGAGAGGTCTGGCTGTAGA-3′ and 5′-GCGTCGGCTTTCTGTTTCA-3′, *Danio rerio* IL-8*b*, 5′-TGTTTTCCTGGCATTTCTGACC-3′ and 5′-TTTACAGTGTGGGCTTGGAGGG-3′.”

