# Supplementary material for: Treponema pallidum (syphilis) antigen TpF1 induces angiogenesis through the activation of the IL-8 pathway
Source: Sci Rep. 2016 Jan 5;6:18785. doi: 10.1038/srep18785 (PMC4700457; doi:10.1038/srep18785)
Supplement: Supplementary Information 1 [file srep18785-s1.pdf]

Supplementary material

Treponema pallidum (syphilis) antigen TpF1 induces angiogenesis through the activation of the IL-8 pathway

Running title: TpF1 stimulates angiogenesis

Tommaso Pozzobon[1#], Nicola Facchinello[1#], Fleur Bossi[2#], Nagaja Capitani[3,4], Marisa Benagiano[4], Giulietta Di Benedetto[5,6], Cristina Zennaro[2], Nicole West[2], Gaia Codolo[1], Marialina Bernardini[7], Cosima Tatiana Baldari[3], Mario Milco D'Elios[4], Luca Pellegrini[8], Francesco Argenton[1*], Marina de Bernard[1*]

[1] Department of Biology, University of Padua, Padua, Italy

[2] Department of Medicine, Surgery and Health Sciences, University of Trieste, Trieste, Italy

[3] Department of Life Sciences, University of Siena, Siena, Italy

[4]Department of Experimental and Clinical Medicine, University of Florence, Florence, Italy

[5] Institute of Neuroscience, Italian National Research Council (CNR), Padua, Italy

[6] Venetian Institute of Molecular Medicine, Padua, Italy

[7] Department of Biology and Biotechnology, Sapienza University of Rome, Rome, Italy

[8] Department of Molecular Biology, Medical Biochemistry and Pathology, Université Laval, Quebec, Canada

[#] T. Pozzobon, N. Facchinello and F. Bossi equally contributed to this paper

***Corresponding authors**

Francesco Argenton, francesco.argenton@unipd.it

Marina de Bernard, marina.debernard@unipd.it

Supplementary Figures

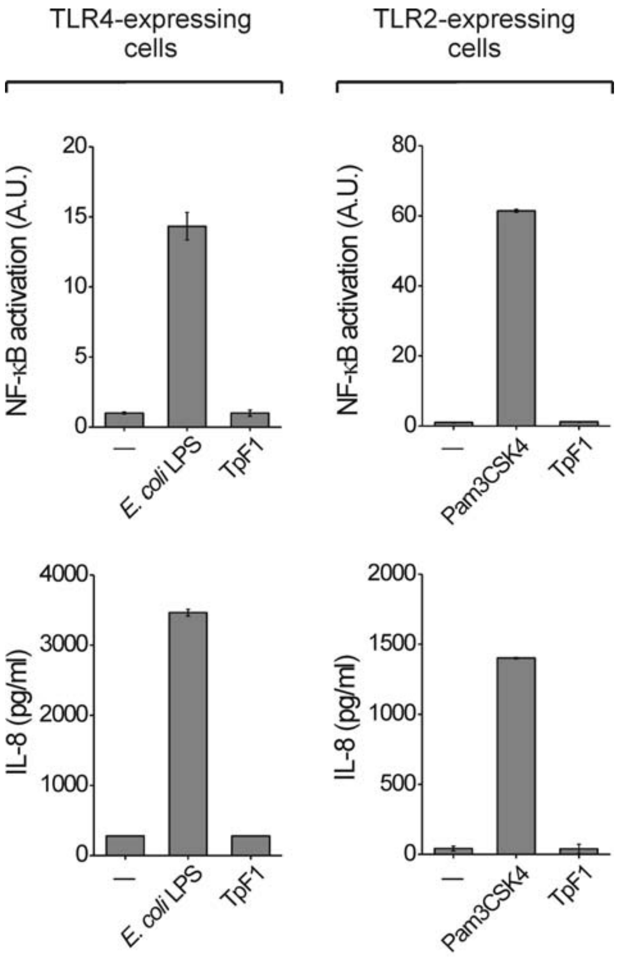

Figure S1. TpF1 preparations are free from Gram-negative and Gram-positive contaminants. HEK-293 cells expressing human Toll-Like-Receptor 4 (hTLR4)/MD2/CD14 and HEK-293 cells expressing human Toll-Like-Receptor 2 (hTLR2) were challenged with TpF1 (100 ng/ml) and, as positive controls, with *E. coli* LPS (100 ng/ml) and Pam3CSK4 (1 µg/ml). After 4 h (hTLR4) and 6 h (hTLR2), NF-κB activation was measured using the luciferase reporter assay system, and normalized to that of non-stimulated cells. Luciferase activity values from unstimulated cells were set at 1 arbitrary unit (A.U.). IL-8 production was quantified after 18 h stimulation with the specified agonists. Data are the mean values obtained from three independent experiments repeated twice.

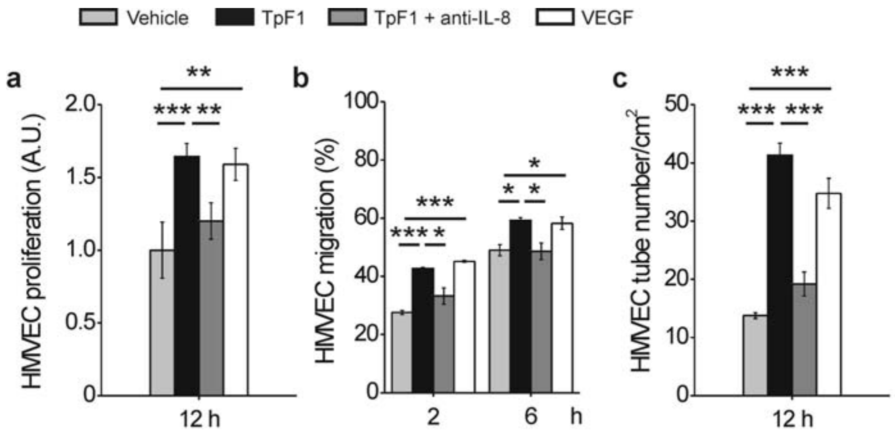

Figure S2. TpF1 induces IL-8-dependent proliferation and migration of dermal microvascular endothelial cells and their organization in microcapillary-like structures. (**a**) HMVEC-D were exposed to TpF1 (in the presence or absence of anti-IL-8 blocking antibody), VEGF or vehicle. After 12 h, cells were counted and normalized to the number of plated cells set as 1 arbitrary unit (A.U.). The graph shows cell proliferation under the different conditions. Data are represented as the mean ± S.D. of three independent experiments. (**b**) HMVEC-D, grown to confluence in 6-well plates, were exposed to TpF1 for 24 h. Supernatants were collected and incubated or not with anti-IL-8 blocking antibody for 45 min, before transferring to the lower chamber of 24 Transwell plates with Fast DiI-labelled HMVEC-D seeded on the upper chamber. VEGF and vehicle added to the lower chamber were used as positive and negative control, respectively. Migrated cells were quantified after 2 and 6 h. Data are represented as the mean ± S.D. of three independent experiments. (**c**) HMVEC-D were seeded on Matrigel-coated coverslips and exposed to TpF1 (in the presence or absence of anti-IL-8 blocking antibody), VEGF or vehicle, for 12 h. Cells were stained with Phalloidin-Alexa Fluor 546 and analyzed by confocal microscope. The number of tubes formed was counted and expressed as number of tubes/cm^2.
